# Perceived self-efficacy and willingness to teach family planning among nursing and midwifery faculty in higher learning institutions in Rwanda

**DOI:** 10.1186/s12909-023-04941-7

**Published:** 2023-12-20

**Authors:** Aimable Nkurunziza, Madeleine Mukeshimana, Tamrat Endale, Michael Habtu, Yvonne Delphine Nsaba Uwera, Reverien Rutayisire, Justine Bagirisano, Jean Bosco Henri Hitayezu, Marie Laetitia Bazakare Ishimwe, Jean de Dieu Uwimana

**Affiliations:** 1 Arthur Labatt Family School of Nursing, Faculty of Health Sciences, London, Canada; 2 School of Nursing and Midwifery, College of Medicine and Health Sciences, University of Rwanda, Kigali, Rwanda; 3 The Center for International Reproductive Health Training, University of Michigan, (CIRHT-UM), Michigan, USA; 4 School of Public Health, College of Medicine and Health Sciences, University of Rwanda, Kigali, Rwanda; 5 Single Project Implementation Unit (SPIU), University of Rwanda, Kigali, Rwanda; 6 Center for Teaching and Learning Enhancement(CTLE), University of Rwanda, Kigali, Rwanda; 7 Lawrence Bloomberg Faculty of Nursing, University of Toronto, Toronto, Canada; 8 School of Health Sciences, College of Medicine and Health Sciences, University of Rwanda, Kigali, Rwanda

**Keywords:** Self-efficacy, Family planning, Nursing, Midwifery, Faculty, Teaching, Higher learning institution

## Abstract

**Background:**

Promoting family planning (FP) is a key strategy for health, economic and population growth, and achieving sustainable development goals (SDGs) especially SDG 3, which promotes health and well-being for all. The quality of FP services depends on the training of competent nursing and midwifery graduates before entering the workforce. In order to ensure graduates are well-trained and capable of meeting the needs of the population, their teachers need to demonstrate high self-efficacy and willingness to teach FP. However, there is a lack of research on the capacity and willingness of nursing and midwifery faculty to teach FP at higher learning institutions (HLIs) in Rwanda. The objective is to investigate and articulate the perceived self-efficacy and willingness of the nursing and midwifery faculty to instruct HLIs students on FP.

**Research design/Methodology:**

We conducted a mixed methods study using a sequential explanatory design among almost all the HLIs (n = 6, 1 institution declined) that train nurses and midwives in Rwanda. One hundred thirty-six nursing and midwifery faculty who were actively teaching FP either in class, simulation lab, or clinical practice were invited to participate in a self-administered questionnaire and four qualitative focused group discussions. Participants answered questions ranking their self-efficacy in four domains from 0 - not confident to 3 - completely confident. Scores were calculated for each domain. A semi-structured interview guide was developed based on quantitative survey findings to gain a deep understanding of the ability and willingness to teach FP. Data were analyzed using thematic analysis. Ethical approval was obtained from the University of Rwanda, College of Medicine and Health Sciences Institutional Review Board.

**Results:**

A total number of 89 nursing and midwifery faculty participated in the study and only 85 completed the questionnaires fully, yielding a response rate of 95.5%. The mean age was 40.39 and there were more females (62.4%) compared to their male counterparts (37.6%). Respondents scored highest for perceived self-efficacy in course preparation (mean = 2.37), evaluation and examination (mean = 2.12) and instructor behavior and delivery (mean = 2.35). However, the score was low for clinical practices (mean = 1.79). There was a significant correlation among the four items of self-efficacy (p < 0.05). Being a female, a midwife, and having more years of experience in nursing education were each significantly associated with perceived self-efficacy to teach FP (p < 0.05). In the qualitative phase, 32 study participants participated in four focus group discussions. Four themes were identified: (a) educational background as a determinant of confidence to teach FP; (b) willingness to teach FP; (c) enabling factors of teaching FP; and (d) structural challenges.

**Conclusion:**

Nursing and midwifery faculty reported inadequate self-confidence in teaching FP in clinical practice. Addressing personal and structural challenges in teaching FP should be a top priority. This requires a collective effort between nursing and midwifery faculty and HLIs to dismantle individual and systemic barriers that hinder self-efficacy and willingness to teach FP. There is a need for HLIs and different stakeholders to invest in training the nursing and midwifery faculty on FP practical skills to have a nursing and midwifery workforce providing up-to-date clinical FP services that will help Rwanda reach the SDGs.

**Supplementary Information:**

The online version contains supplementary material available at 10.1186/s12909-023-04941-7.

## Introduction

Family Planning (FP) is a key strategy for health, economic and population growth, and to achieve sustainable development goals (SDGs), especially SDG 3, which promotes health and well-being for all [1, 2]. SDG target 3.7 ensures that sexual and reproductive health services, including FP, information, and education, are widely accessible, and that reproductive health is integrated into national programs and strategies [3]. Unfortunately, unmet needs for FP continue to be worrisome in developing countries, including Sub-saharan Africa (SSA). About 218 million women in these countries have unmet contraception needs, i.e., they want to prevent unintended pregnancy but they don’t use a modern contraceptive. Approximately half (49%) of pregnancies in low and middle income countries (LMICs) are unintended, amounting to 111 million pregnancies each year [4]. The common reasons for not using contraceptives are fear of side effects and health risks, having sex infrequently or not at all, family members’ disapproval, breastfeeding, and/or amenorrhea [5, 6]. If women in LMICs had access to modern contraception and received adequate prenatal care, unintended pregnancies and unplanned births would decrease by 68% [4]. Moreover, unsafe abortions would decrease by 72% and 113,000 maternal deaths could be prevented annually, a reduction of 62% in maternal mortality [4].

FP is an effective and inexpensive solution for reducing maternal mortality in high-fecundity countries [1], including developing countries. For instance, various countries have reported reductions in maternal mortality among women through FP ranging from 6 to 60% [7]. However, the low knowledge and skills of healthcare providers leads to poor quality of care in FP services [8, 9]. These deficits in provider knowledge and skills can be attributed to poor pre-service education. Findings from program evaluation and research revealed that students in traditional medicine, nursing and midwifery education programs do not receive adequate FP training and could not provide these services as soon as they entered the field [8, 10]. Moreover, training irregularities manifest themselves in clinical practice when recently trained clinicians lack the necessary practical skills to provide quality and evidence-based FP services [8, 10, 11]. Educating health workers before they enter the workforce is essential for developing their skills and knowledge [10, 12, 13], and pre-service training is key in improving the current updates. Including FP training in pre-service medical and nursing programs is an effective and cost-efficient way of enabling new clinicians to provide quality and evidence-based FP services [14–16].

Evidence suggests that clinicians need training in FP that corresponds with the cultural context and the every-day realities of clinical practice. For example, it has been shown that nurses and midwives do not have enough knowledge and skills to provide long term and permanent contraceptive methods [9, 17] and fertility awareness-based methods, including natural FP methods [18]. According to a recent study by Schwandt and colleagues [17] conducted in Rwanda, the most commonly used modern contraceptive methods in Rwanda were injectables, at a rate of 41%, followed by condoms at 22%, and implants at 19%. In another study conducted by Mukamuyango et al. [19] and which was focusing on long-acting reversible contraceptives (LARC), only 37% of the participants were using Jadelle, a 5-year progestin-based implant, while 26% were using Implanon, a 3-year progestin-based implant, and 11% were using copper intrauterine device. This low uptake of LARC can be attributed to the lack of training, insufficient confidence, and inadequate counselling skills of healthcare providers [20]. Although several factors influence FP usage, these statistics illustrate a need for clinician training in all the available up to date modern contraceptive methods and FP strategies to ensure new clinicians provide quality of care and meet the population needs when they enter the labor market. In order to ensure graduates are well-trained and capable of meeting the needs of the population, their teachers’ self-efficacy and willingness to teach FP need to be assessed.

According to Bandura [21], as a person’s professional career progresses self-efficacy may change. Scholars found that nurse educators develop efficacy through time and with greater teaching experience [22, 23]. Several studies in other countries/regions outside Rwanda have found that nurse educators’ self-efficacy is influenced by clinical experience [24–28], formal education, on-the-job training, and continuous professional development [22, 28–30], as well as, teaching aids and infrastructure, including well-equipped simulation labs [8, 14]. Moreover, cultural beliefs about FP may impact teacher self-efficacy among other factors [31].

Nursing and midwifery training in Rwanda follows different paths based on institution-specific guidelines. Both nursing and midwifery programs offer direct entry into postsecondary institutions (advanced diploma for three years and bachelor’s degree for four years) and a bridging program from advanced diploma to bachelor’s degree for two years. Graduates of these programs are registered nurses or registered midwives with the same scope of practice and additional nursing research that bachelor’s degree holders receive [32]. In these programs across the county, the FP content was not harmonized and was not competency-based [10] and this could impact how nursing and midwifery faculty self-efficacy and willingness to teach FP. However, the literature from Rwanda on nursing and midwifery educators’ self-efficacy in teaching FP is scarce. The authors conducted this study to fill a gap in our understanding of how pre-service FP and contraception education impact the practice of midwives and nurses once they are deployed in different health facilities. Since the existing ways of teaching contraception and FP among nursing and midwifery students could impact future service delivery in Rwanda, this study asked nursing and midwifery faculty to self-identify training gaps and reflect on their efficiency in teaching those subjects. Thus, this study aims to describe the perceived self-efficacy and willingness of the HLIs nursing and midwifery faculty to teach contraception and FP as part of pre-service training programs with the aim of improving the quality of clinical service delivery among new graduates.

## Methods

### Design

To explore the teaching of FP from diverse perspectives and to provide a more comprehensive analysis, we employed a mixed-method design [33]. Researchers utilized a sequential explanatory study design to elucidate intricate relationships and patterns among variables, leading to a deeper understanding of how FP is taught [33]. In the initial quantitative research phase, the findings yielded an overarching understanding of FP instruction, guiding the development of the interview guide and the selection of participants for the subsequent qualitative phase. The quantitative phase utilized a descriptive cross-sectional design, while the qualitative phase employed a descriptive exploratory design to gain an in depth understanding of nursing and midwifery faculty’s perceived self-efficacy and willingness to teach FP.

### Study population and setting

A total population sampling employed to enlist all nursing and midwifery faculty members from every HLIs in Rwanda. HLIs staff actively engaged in administrative and part-time roles were not included in the study. A total of 136 nursing and midwifery faculty who are actively teaching students in classroom, simulation lab, and in the clinic setting in seven HLIs in Rwanda were invited to participate in the study. Among these, one public and six private HLIs in Rwanda are accredited to provide training for nurses and midwives, offering advanced diploma and/or bachelors’ degree programs. Notably, three of these institutions are situated in urban areas, while the remaining four are located in rural areas [34]. One urban HLI declined the involvement of its staff, and other employees from one private rural HLI took part in the pilot study of the quantitative instrument and the subsequent qualitative phase. Thus, a total number of 119 study participants were identified eligible to participate. Out of these, fifteen study participants were enlisted for the instrument pilot were excluded from the final sample. Invitations were extended to 104 participants from five HLIs, with 89 consenting to participate; however, four questionnaires were incomplete. Hence, this study focused on the data from 85 participants.

### Data collection instruments

In the quantitative phase, the Self-Efficacy Towards Teaching Inventory for Nurse Educators (SETTI-NE) [35], an individual self-assessment survey, was customized and subjected to a pilot study with the author’s permission. This instrument tool comprises two sections: the first section features contextualized socio-demographic questions, while the second section encompasses 54 items gauging self-efficacy across course preparation (10 questions), instructor behavior and delivery (14 questions), evaluation and examination (14 questions), and clinical practice (16 questions). The items underwent randomization and were assessed using a four-point scale: not confident, somewhat confident, moderately confident, and completely confident. Each question within each component received a score on a 0–3 scale. The average score for each of the four domains was obtained by averaging the scores of the individual questions within that component. The questionnaires were electronically completed through KoboToolbox. Adjustments were made to the demographic section, incorporating new items while removing others. Notably, positions such as assistant clinical professors, associate clinical professors, or clinical professors were excluded, reflecting their absence in Rwanda’s HLIs. Conversely, a tutorial assistant and an assistant lecturer were included. Moreover, the original tool exclusively discussed nursing; hence, midwifery components were incorporated to align with the study population. Additionally, our study deemed it necessary to include the institution’s type (public or private), location (urban or rural), and religious affiliation. Questions related to the effectiveness of teaching skills and behaviors remained unchanged. A pilot study involving 15 study participants yielded an overall Cronbach’s Alpha of 0.975. The reliability test for each SETTI-NE component included self-efficacy and ability in course preparation (0.914), self-efficacy in instructor behavior and delivery (0.919), self-efficacy in evaluation and examination (0.950), and self-efficacy in clinical practice (0.979). Utilizing insights from the quantitative phase, we developed a semi-structured focus group interview guide to explore the willingness of nursing and midwifery faculty to teach FP.

A qualitative interview guide, crafted by experienced nursing and midwifery educators and approved by senior researchers, was employed in the study. The following invitation questions were utilized:

1. Tell me how you feel when teaching FP.
2. Tell me about the challenges you face when teaching FP.
3. What factors influence your ability to teach FP?
4. What factors hinder your ability to teach FP in classrooms, simulation labs, and clinical settings?
5. How do personal beliefs impact your ability to teach FP?
6. What do you think can improve your teaching of FP?
7. What else would you recommend to improve teaching FP?

### Data collection procedures

The study was initiated after obtaining approval from the relevant authorities and securing informed consent from the study participants. A team of five members was present on-site to address any inquiries from study participants and assess adherence to inclusion criteria. Prior to questionnaire distribution, participants received comprehensive information about the the study’s purpose and inclusion criteria. The research team conveyed this information in staff meetings by reading an informational letter to the participants. Study participants were explicitly informed about the voluntary nature of their involvement. As part of the study reporting, study participants were also informed that the study would maintain confidentiality and not disclose their identities or school names. Each participant provided their email to receive the data collection link. Data collection was conducted using an online tool called KoboToolbox. Study participants spent approximately 30 to 45 min completing the questionnaire. Upon submission of the questionnaire, each participant was remunerated with 3,000 Rwandan Francs (equivalent to approximately $2.45) for internet bundle usage. The data collection period spanned from July to August 2022.

Towards the conclusion of the quantitative survey, participants were asked if they wished to partake in the qualitative phase of the study. Based on their responses, four focus groups (FDGs) were formed, each comprising ten participants with diverse institutional and professional backgrounds. The moderators for the FGDs were members of the research team and experienced midwives with qualitative interviewing proficiency. Before commencing the interviews, the moderators established a rapport with the participants, elucidating the study’s nature and the interview process while securing informed consent. Permission for recording was also obtained, and participants were assured of the confidentiality of their data. To ensure privacy, identifiers were removed, creating a secure dataset accessible only to research team members. Study participants were designated with codes (MD: midwife respondent and NS: nurse respondent). The interviews took place in a private and quiet room.

Given that all participants were proficient in English, data collection was conducted in English. The average duration of the focus group interviews was 1 h and 16 min. Subsequent to the interview, each participant received 10,000 Rwandan Francs (approximately $8.17) to cover their time, transportation, and communication expenses.

### Ethical considerations

The study obtained ethical clearance from the University of Rwanda College of Medicine and Health Sciences Institutional Review Board (IRB) No 335/CMHSIRB/2022. Subsequent to this approval, the researchers sought permission from all HLIs that provide training for nurses and midwives. Upon securing permissions from the respective institutions, the researchers approached potential study participants, elucidating the standards of informed consent, and outlining the associated risks and benefits. Those who were willing to participate signed a written consent form and received a modest compensation to cover their time, transportation, and communication expenses. All data are securly stored in a locked computer accessible exclusively to the research team. The data will be discarded in accordance with the ethical guideliness set forth by the University of Rwanda.

### Analysis

For the quantitative phase, data were extracted from KoboToolbox and imported to SPSS version 26. Descriptive analysis encompassed frequencies, percentages, means and standard deviation. A scoring system was employed for the self-efficacy components assessed on a scale with scores as follows: 0 for *‘not confident’*; 1 for*‘somewhat confident’*, 2 for*‘moderately confident’* and 3 for *‘completely confident’*. The overall average was computed by consolidating the items in each group. A Pearson correlation coefficient was employed to evaluate the relationship among the various self-efficacy components. Relationships between sociodemographic variables and faculty-perceived self-efficacy were determined through independent *t*-test and one-way ANOVA, comparing the mean score for each component. The Significance level was set at *p*-value 0.05.

In the qualitative phase, verbatim transcriptions of FGDs were conducted, and Dedoose was utilized for data organization. Thematic analysis, involving six steps [36] (data familiarization, generating initial codes, searching for themes, reviewing themes, defining themes, and writing up), was performed by four research team members. The team developed and refined codes through consensus and iteratively reviewed them. Connections between codes led to the identification of analytical categories and an overarching explanation. Several techniques were employed to enhance data trustworthiness: the research team members who conducted the FGDs also conducted data analysis; the entire research team checked and refined the coding categories, data collection and analysis occurred concurrently to inform one another iteratively, and references to the literature were consistently incorporated [37].

## Results

### Quantitative data

#### Socio-demographic information of respondents

A total of 85 participants from nursing and midwifery faculty were involved in this study. The participants’ average age was 40.39 years (n = 85), with 54.1% (n = 46) falling in the 30 to 39 years age group. There majority of respondents were female (62.4%; n = 53), while 37.6% (n = 32) were male. A significant proportion of participants taught at public institution (72.9%; n = 62) and resided in urban areas (80.0%; n = 68). The majority (75.3%; n = 64) held a Master’s degree, and their primary professional background was predominantly in nursing (60.0%; n = 51). Most participants (63.5%; n = 51) held the position of assistant lecturers, and over a third (37.6%; n = 32) had accumulated 11 to 15 years of experience in nursing or midwifery education. However, a majority of participants (55.3%; n = 47) had less than six years experience in a clinical setting. Regarding religious affilations, majority (72.9%; n = 62) were associated with mixed-religion institutions (Table 1).

**Table 1:** Sociodemographic information of respondents (N = 85)

Variables	n = 85	%	
**Age [years]**			
30 to 39	46	54.1	
40 and above	39	45.9	
Mean (SD) = 40.39(7.47)			
**Gender**			
Male	32	37.6	
Female	53	62.4	
**Type of institution**			
Public institution	62	72.9	
Private institution	23	27.1	
**Residence/locality**			
Urban	68	80.0	
Rural	17	20.0	
**Level of education**			
Bachelor’s degree	21	24.7	
Master’s degree	64	75.3	
**Educational background**			
Midwife	34	40.0	
Nurse	51	60.0	
**Academic Rank**			
Tutorial assistant	27	31.8	
Assistant lecturer	54	63.5	
Lecturer	4	4.7	
**Work experience in nursing/midwifery education**			
< 6 years	10	11.8	
6 to 10 years	29	34.1	
11 to 15 years	32	37.6	
> 15 years	14	16.5	
**Work experience in clinical setting**			
< 6 years	47	55.3	
6 to 10 years	16	18.8	
> 10 years	22	25.9	
**Teaching experience in other fields**			
Yes	48	56.5	
No	37	43.5	
**Participant’ institution according to religious identity**			
Protestant	18	21.2	
Catholic	5	5.9	
Non-religious	62	72.9	

### Perceived self-efficacy across four domains among respondents (N = 85)

The comprehensive breakdown of each component regarding perceived self-efficacy in teaching FP can be found in Additional File 1. Participants demonstrated a high average score of perceived self-efficacy in course preparation (mean = 2.37) and instructor behavior and delivery (mean = 2.35). Conversely, the score was comparatively lower for clinical practices (mean = 1.79), as indicated in Additional File 1.

### The correlation between socio-demographic characteristics and perceived self-efficacy in teaching FP

Notably, increased work experience exhibited a significant association with self-efficacy in course preparation (F = 5.07; p = 0.003). Similarly, experience in nursing/midwifery education (F = 6.15; p = 0.001) and clinical work experience (F = 3.20; p = 0.046) demonstrated significant correlations with self-efficacy in instructor behavior and delivery.

The mean score for self-efficacy in evaluation and examination was notably higher among midwives (t = 3.59; p = 0.001) and those with over ten years of experience in teaching nursing/midwifery (F = 2.90; p = 0.040). Significantly, being female (t = -2.12; p = 0.038), trained as a midwife (t = 5.03; p < 0.001), and having more years as a nurse educator (F = 3.23; p = 0.027) were associated with perceived self-efficacy to teach FP. However, the type of institution and locality did not show significant differences in FP teaching self-efficacy **(**Table 2**)**.

**Table 2:** Relationship between socio-demographic characteristics and perceived self-efficacy across four domains among respondents (N = 85)

Variables	Course preparation			Instructor behavior and delivery			Evaluation and examination			Clinical practice	
	Mean (SD)	*p* value		Mean (SD)	*p* value		Mean (SD)	*p* value		Mean (SD)	*p* value
**Age [years]**											
30 to 39	2.39(0.46)	0.735		2.32(0.44)	0.494		2.00(0.76)	0.088		1.73(0.99)	0.547
40 and above	2.35(0.49)			2.39(0.49)			2.27(0.64)			1.86(0.89)	
**Gender**											
Male	2.45(0.52)	0.268		2.40(0.50)	0.447		1.93(0.84)	0.056		1.52(1.08)	**0.038**
Female	2.33(0.45)			2.32(0.45)			2.24(0.61)			1.96(0.83)	
**Type of institution**											
Public	2.41(0.40)	0.266		2.38(0.40)	0.486		2.20(0.68)	0.121		1.91(0.89)	0.068
Private	2.29(0.64)			2.29(0.63)			1.93(0.79)			1.48(1.04)	
**Residence**											
Urban	2.42(0.42)	0.066		2.38(0.42)	0.247		2.09(0.74)	0.411		1.77(0.97)	0.66
Rural	2.18(0.64)			2.24(0.63)			2.25(0.62)			1.88(0.87)	
**Level of education**											
Bachelor’s degree	2.24(0.44)	0.15		2.25(0.38)	0.287		2.04(0.68)	0.545		1.68(0.97)	0.534
Master’s degree	2.42(0.48)			2.38(0.49)			2.15(0.73)			1.83(0.94)	
**Educational background**											
Midwife	2.41(0.40)	0.573		2.40(0.38)	0.45		2.44(0.41)	**0.001**		2.35(0.42)	**< 0.001**
Nurse	2.34(0.52)			2.32(0.52)			1.91(0.79)			1.42(1.02)	
**Academic Rank**											
Tutorial assistant	2.30(0.45)	0.609		2.35(0.42)	0.638		2.19(0.68)	0.273		1.78(0.94)	0.498
Assistant lecturer	2.40(0.48)			2.34(0.49)			2.13(0.74)			1.83(0.96)	
Lecturer	2.37(0.47)			2.57(0.46)			1.57(0.51)			1.25(0.96)	
**Work experience in nursing/midwifery education**											
< 6 years	2.07(0.51)	**0.003**		2.08(0.45)	**0.001**		2.06(0.62)	**0.04**		1.81(0.87)	**0.027**
6 to 10 years	2.28(0.50)			2.28(0.53)			1.84(0.81)			1.38(1.00)	
11 to 15 years	2.39(0.42)			2.32(0.37)			2.28(0.59)			2.01(0.81)	
> 15 years	2.74(0.29)			2.77(0.29)			2.40(0.67)			2.12(0.97)	
**Work experience in clinical setting**											
< 6 years	2.29(0.53)	0.088		2.27(0.49)	**0.046**		2.02(0.73)	0.309		2.07(0.54)	0.129
6 to 10 years	2.59(0.34)			2.61(0.33)			2.32(0.71)			2.37(0.50)	
> 10 years	2.38(0.39)			2.34(0.44)			2.19(0.68)			2.20(0.95)	
**Teaching experience in other fields**											
Yes	2.38(0.49)	0.785		2.40(0.48)	0.286		2.03(0.75)	0.192		1.69(1.0)	0.291
No	2.35(0.46)			2.29(0.45)			2.24(0.66)			1.92(0.86)	
**Participant’ institution according to religious identity**											
Protestant	2.26(0.69)	0.513		2.24(0.67)	0.503		1.86(0.78)	0.216		1.31(0.95)	0.051
Catholic	2.34(0.43)			2.47(0.42)			2.16(0.88)			2.08(1.21)	
Non-religious	2.41(0.39)			2.37(0.40)			2.20(0.67)			1.91(0.89)	

Bolded *p* values indicate significant association

Independent *t* test was used to compare means of two categories while one way ANOVA was used to compare means for more than 2 categories

### Qualitative results

Overall, the majority of respondents indicated that they taught FP in either a classroom, a simulation lab, or clinical settings, or a combination of these. However, when questioned about their sentiments concerning their perceived ability and willingness to teach FP, the respondents conveyed mixed feelings. Four discernible themes emerged: (a) Educational background as a determinant of confidence to teach FP; (b) Willingness to teach FP; (c) Enabling factors of teaching FP; and (d) Structural challenges.

#### Educational background as determinant of confidence to teach FP

Certain participants in the study, drawing from their experience, expressed confidence in teaching both natural and hormonal FP methods. For instance, one study participant stated: *“I feel confident when I am teaching FP as I have been trained for many years and have experience in both clinical and teaching areas. I feel a bit confident when I’m teaching the students***” (**MD06). Another respondent (MD02) affirmed, **“***I feel very confident to teach FP in HLIs because I have five years’ experience in teaching this course, which is why I am very comfortable teaching FP content*”.

While many respondents expressed confidence, a few mentioned being confident only in teaching natural methods, and some admitted to lacking confidence altogehter. For example, one participant stated: “*For me, when I am teaching in FP, I am confident in some methods, not all methods… I am not really confident, for example, teaching Implanon or Jadelle…”*, **(**NS03). Similarly, another participant emphasized: *“Related to teaching FP, I need more knowledge… because sometime[s] there are some FP methods I have confusion about. The reason why is I need to get more clarification and knowledge*.” (NS01) A similar concern was raised by another participant who said:

*While I have a strong grasp of the theoretical components, my practical exposure is not comprehensive across all FP methods. For instance, I am proficient in teaching natural methods such as the use of calendar, but my confidence wavers when it comes to methods like insertion of implants and IUD.* (NS06).

Midwives conveyed a strong sense of confidence in their ability to teach FP, whereas nurses reported a lower level of knowledge and skills, particularly in instructing certain invasive FP methods.

#### Willingness to teach FP

Some respondents conveyed a positive perception and willingness to teach FP, emphasizing its status as a human right and its critical role in reducing maternal morbidity and mortality. Intriguingly, individuals from both nursing and midwifery backgrounds indicated that they could solely teach natural FP theory and skills, while others explicitly stated their reluctance to teach FP altogether. They emphasized a preference for others to deliver the course, citing a lack of belief in FP utilization or provision. A nurse participant expressed: *“I feel uncomfortable to deliver FP course because of my beliefs …… I do not believe in FP "* (NS10). Similarly, a midwife participant added: *“…while I am teaching FP methods, I have judgement of those artificial methods because, based on my beliefs, I believe that using those FP methods is committing a sin…” (*MD14). However, another participant presented a different perspective on teaching FP:

*When it comes to teaching FP, I approach it without being judgmental. I hold no opinions about any FP methods because, in my understanding, FP is a human right; it is entirely voluntary, not an obligation. My approach is to provide comprehensive information and skills. Student can then choose to adopt and apply this information and skills.* (NS14)

The quotes above indicate that the manner in which FP is taught varies depending on the instructor’s beliefs. Some instructors abstain from teaching artificial methods due to judgmental attitudes stemming from their religious beliefs.

#### Enabling factors of teaching FP

Several participants in the study emphasized their ability to teach FP owing to the knowledge and skills acquired through various trainings, access to infrastructure, and clinical exposure. Two participants expressed their confidence: *“Hmm…I feel confident to teach FP because of materials, the equipped skills laboratory, and also skills and knowledge I gained in various FP trainings”* (MD11) and NS15). Another participant (NS07) stated: *“I have been trained about different FP methods.”* She further elaborated on her motivation: *“I feel motivated to help these young mothers who have an issue of unplanned pregnancies and the problems they face.”* These quotes underscore that a combination of factors contributes to the cultivation of self-efficacy in teaching FP.

#### Structural challenges

The respondents outlined various challenges associated with their institutions, including: a lack of training in the new FP methods introduced in the country; limited exposure to FP methods in clinical settings; absence, insufficiency, or malfunctioning of teaching materials, especially anatomical models; inadequate infrastructure; increased workload, and shortage of time allocated to FP teaching in both theory and practice. A nurse faculty member (NS15) highlighted, *“Barriers are the lack for materials…Most of the staff in academic are not trained on new methods introduced in the country.”* Another participant (NS01) explained how the teacher-student ratio poses a challenge to FP teaching: “*…when there is a large class with a significant number of students, because it is a practical module, it won’t be easy to demonstrate to all these students to ensure that they are acquiring the knowledge and skills required in FP.”* In a similar vein, another participant emphasized, *“Checklists and course syllabuses are organized, but there is a need to be always updated according to the needs and new guidelines and protocols”* (MD08).

Several respondents expressed shared concerns regarding the practice of some faith-based schools of nursing and midwifery, which predominantly send their students for clinical practice to faith-based healthcare institutions where patients lack access to FP services as a nurse faculty member (NS12) explained in the following quote:

*The school itself, frankly speaking, is a religious-based school. Previously, they wanted us to focus more on natural methods only. As teachers, we didn’t really complain before, but now I am proud to be part of teaching as required. We have been trained about these modern methods, and now we are teaching both natural and modern contraceptive methods.*”

## Discussion

This study investigated the perceived self-efficacy and willingness of the nursing and midwifery faculty to teach FP to students at HLIs in Rwanda. Within the study framework, self-efficacy was defined as the belief in an individual’s ability to perform specific behaviours through four ways or processes: cognitive, motivational, affective, and selection [38, 39]. The assessment of perceived self-efficacy in teaching FP in this study encompassed specific and pertinent components, including: self-efficacy and ability in course preparation; self-efficacy in instructor behaviour and delivery; self-efficacy in evaluation and examination; and self-efficacy in clinical practice [35].

The current study reveals that self-efficacy for teaching FP among respondents, particularly in clinical practice, is insufficient. While participants exhibited a high average score for perceived self-efficacy in course preparation and instructor behavior and delivery, their self efficacy was notably lower for clinical practices. The self-efficacy of educators can vary based on the specific content they teach, with potential discrepancies between high self-efficacy in lab simulations and lower confidence in lecturing [40]. Study participants expressed a lack of confidence in practice due to insufficient exposure to practical skills. These findings align with a study in Tanzania, which identified that nurse educators feel more confident in teaching theory than practice, citing a lack of exposure to practices and the absence of FP clinics attached to their training schools [14]. It is widely acknowledged that clinical experience enhances self-efficacy in teaching nursing and midwifery procedures [26]. However, contradictory evidence exists, as one study found no correlation between clinical experience and the development of self-efficacy [29].

In this study, increased work experience was significantly linked to self-efficacy in FP course preparation. The more years of experience teaching FP a faculty member possesses, the higher their self-efficacy in teaching FP tends to be. As one’s career progresses, self-efficacy may undergo changes [21]. Prior research has indicated that nurse educators develop efficacy over time and through teaching experience [22, 23]. The study identified that experience in a clinical setting was significantly correlated with self-efficacy in instructor behavior and delivery, in line with findings from other studies [26–28]. According to Bandura [41], practicing skills leads to performance accomplishments, contributing to an increase in self-efficacy. In Tanzania, nurse educators attributed the lack of practical exposure as the primary cause of producing graduates with insufficient skills to practice FP [14]. Additionally, having extensive experience in nursing and midwifery education was significantly associated with self-efficacy in instructor behavior and delivery. This aligns with existing litreature indicating that clinical instructors with formal nursing education or through orientation to their role may function effectively [22, 28–30].The study’s results underscore the specific need for enhancing FP teaching efficacy, especially among nurses and midwives lacking clinical exposure to FP and formal competency-based FP training.

Regarding self efficacy in educational evaluation and examination, respondents with a midwifery background and those with more than ten years of teaching experience had significantly higher mean scores compared to respondents with a nursing backgroud and less experience, respectively. In the qualitative segment of this study, respondents with a nursing background expressed a lack of confidence in teaching long acting FP methods or procedural skills, such as insertion of implants and intrauterine devices (IUDs), while respondents with a midwifery background reported confidence in all methods. This aligns with another study indicating that nurse educators are more at ease teaching FP theory than practice [14]. These findings resonate with results from two studies in Rwanda, which highlighted that nurses lack sufficient knowledge and skills to provide long-acting reversible contraception (LARC) [9, 42]. This association might be attributed to the prolonged exposure of midwives to midwifery interventions, including reproductive health, compared to nurses during education and practice.

In our study, being female was significantly associated with perceived self-efficacy to teach FP, although research conducted in a different context found that general self-efficacy among male nursing and midwifery educators was higher than that of female instructors [43]. Some scholars have found that gender doesn’t alter self-efficacy perception in academia [44, 45], while others have reported the opposite [46, 47]. This discrepancy may be linked to socio-cultural factors or discipline. In the current study, individuals with more years of working in nursing and midwifery education exhibited high self-efficacy in teaching FP. A person’s self-efficacy is influenced by a various factors, including mastery experiences, vicarious experiences, verbal persuasion, and their emotional and physiological states [39].

Some respondents conveyed a positive outlook and eagerness to teach FP, emphasizing its significance as a human right and a critical element in reducing maternal morbidity and mortality. Interestingly, a few respondents indicated their capability to impart natural FP theory and skills, while others explicitly stated their decision not teach FP due to personal and religious convictions. A meta-analysis study discovered that healthcare professionals are influenced by diverse biases and misconceptions when delivering FP services [48]. Hence, it becomes crucial to aknowledge and address these biases and misconceptions when assigning nursing and midwifery faculty to various academic activities related to FP teaching.

Many factors, both personal and institutional, influence how nursing and midwifery faculty educate their students. The outcomes of the current study align with prior research conducted in different countries [8, 14, 49]. The study’s results carry several implications for the cultivation of proficient FP professionals and the delivery of high-quality FP services. This, in turn, could have a potential impact on the sexual and reproductive health and rights of the population, as individuals often base their decisions on information provided by nurses and midwives. The self-efficacy components of nursing and midwifery educators, including personal efficacy, may be influenced by their clinical background [24, 25] while teachers’ efficacy may be affected by their educational background or continuous professional development [22, 28, 29].

Some facilitators, including training programs offered by various stakeholders partnering with HLIs, contribute to the development of the self-efficacy among teaching faculty of FP. The literature underscores the significance of continuous professional development in shaping teacher’s self-efficacy [22, 28, 29]. Additionally, some participants emphasized that access to teaching materials and well-equipped skills laboratory are essential facilitators for effective FP instruction. These findings aligns with research conducted in both India and Tanzania [8, 14].

Our study revealed that inadequate or malfunctioning teaching materials, particularly anatomic models, increased workload, and a limited time allocated to FP teaching, negatively impacted teacher self-efficacy. These findings align with a previous study [14] that identified a lack of essential FP teaching materials, such as medical eligibility chcklist (MEC) wheels, in some colleges with simulation labs, influencing teaching approaches [8]. Moreover, our study highlighted shared concerns among respondents regarding faith-based schools of nursing and midwifery sending students for clinical practice to institutions where FP services are unavailable. This observation resonates with another study indicating that faith authorities may pose challenges to those teaching FP [14]. The intricate relationship between faith and FP at various levels, from individual to government, suggests that collaboration between secular actors, faith leaders, and faith-based organizations is crucial for effective FP advocacy [50].

Participants in our study expressed concerns about outdated curricula and academic documents at their institutions, not being updated in line with current guidelines and protocols. Extensive lierature supports the notion that pre-service information is often outdated, diverging significantly from evidence-based current guidelines. Consequentlty, continuous training for new graduates is necessary to align their knowledge with evolving standards for quality of care for service delivery, as they enter the workforce [8, 51]. Simlar challenges have been noted in India, where teaching faculty prefer using standards textbooks over current national protocols and guidelines [8]. This mismatch between theoretical knowledge and real-life protocols is a cause for concern.

The outcomes of this investigation reveal a deficiency in the self-efficacy of nursing and midwifery faculty when it comes to teaching FP in clinical practice. The deficiency suggests that students may not be adequately prepared to deliver FP services with confidence, potentially contributing to higher unmet FP needs among clients. Rwandan HLIs have an opportunity to address this issue by prioritizing FP training for faculty. This could involve establishing partnerships with international organizations, adopting existing FP training programs to the local context, utilizing e-learning platforms, providing on-the-job training in simulation labs, enhancing supervision and mentorship, fostering peer learning and networking, and emphasizing cultural sensitivity and community engagement. Additionally, practicing nurses and midwives with knowledge and skills not aligned with updated FP guidelines should undergo orientation and on-the-job training. Future research endeavors should encompass evaluations of FP-related curricula and simulation lab to ensure comprehensive support for teaching faculty. The insights gained from these assessments can guide HLIs in identifying and overcoming structural barriers affecting faculty self-efficacy and willingness to teach FP.

### Strengths and limitations

The present study has both strengths and limitations that warrant consideration in interpreting the findings. Firstly, it stands as the first extensive investigation conducted in Rwanda to evaluate self-efficacy in teaching FP among nursing and midwifery faculty in HLIs, encompassing both public and private institutions. Second, all HLIs in Rwanda, with the exception of one institution that declined to participate, were included in the study, contributing to a comprehensive representation. Thirdly, the utilization of a mixed-methods study design facilitated a nuanced exploration of how FP is taught in HLIs, offering a more comprehensive and profound understanding of the teaching landscape [33]. Lastly, the study is aligned with existing national and international partnerships aimed at enhancing FP in HLIs curricula. The generated findings serve as valuable insights for these partners to focus their efforts, and concurrently, inform HLIs about key areas of emphasis when establishing partnerships for FP education.

However, our study is not without limitations. First, we did not directly observe the teaching methods employed by the faculty in the classroom, simulation lab, and clinical settings, which could have provided additional insights to complement our survey and qualitative data. The study also did not conduct a comprehensive review of curricula and teaching materials, such as checklists and logbooks. Additionally, the assessessment simulation labs to gauge their adequacy for facilitating FP teaching was not undertaken. Key informants including heads of departments, deans, and centers of learning and enhancement, were not consulted. Furthermore, it is essential to acknowledge the potential for response bias at both institutional and individual levels. The institution that declined to participate might differ systematically from others. At the individual level, the inclusion of only 85 participants in the quantitative analysis out of the reported 104 at participating institutions (as indicated in Table 1) raises the possiblility that individuals with lower self-efficacy may have been less likely to respond. Lastly, social desirability bias could be a factor due to the sensitive nature of the topic under study.

## Conclusion

The nursing and midwifery faculty expressed a moderate level of self-efficacy in teaching FP, particularly in clinical practice. Our finding reveal that the perceived self-efficacy and willingness of nursing and midwifery faculty in HLIs in Rwanda are influenced by several factors, including personal and religious beliefs, insufficient training in new FP methods introduced in the country, limited exposure in clinical settings, inadequate or nonfunctioning teaching materials, a high student-to-facilitator ratio, limited time allocated to FP teaching in both theory and practice. There is a pressing need for HLIs and other stakeholders to invest in training nursing and midwifery faculty in practical FP skills. The investment is crucial to building a competent nursing and midwifery workforce capable of delivering contemporary clinical FP service, contributing to Rwanda progress towards achieving the Sustainable Development Goals (SDGs).

### Electronic supplementary material

Below is the link to the electronic supplementary material.

Additional file 1: Extent and descriptive statistics among the items of FP teaching self-efficacy.

## Data Availability

The data associated with this study are included within the manuscript. The datasets used in the analysis in this study are available from the corresponding author on reasonable request.
