# Supplementary material for: Perceived self-efficacy and willingness to teach family planning among nursing and midwifery faculty in higher learning institutions in Rwanda
Source: BMC Med Educ. 2023 Dec 20;23:984. doi: 10.1186/s12909-023-04941-7 (PMC10734191; doi:10.1186/s12909-023-04941-7)
Supplement: Supplementary file 1 — Additional file 1: Extent and descriptive statistics among the items of FP teaching self-efficacy. [file 12909_2023_4941_MOESM1_ESM.docx]

**Supplementary file 1: Extent and descriptive statistics among the items of FP teaching self-efficacy**

| **Statements** | **NC, n(%)** | **SC, n(%)** | **MC, n(%)** | **CC, n(%)** | **Mean** | ***Std* Dev** |  |
| --- | --- | --- | --- | --- | --- | --- | --- |
| **Self-efficacy and ability in course preparation** |  |  |  |  |  |  |  |
| State goals and objectives clearly | 1(1.2) | 4(4.7) | 40(47.1) | 40(47.1) | 2.40 | 0.64 |  |
| Plan teaching methodologies | 1(1.2) | 2(2.4) | 46(54.1) | 36(42.4) | 2.38 | 0.60 |  |
| Write a course syllabus | 0(0.0) | 6(7.1) | 47(55.3) | 32(37.6) | 2.31 | 0.60 |  |
| Plan discussions (in class or online) | 1(1.2) | 6(7.1) | 40(47.1) | 38(44.1) | 2.35 | 0.67 |  |
| Plan teaching and learning activities | 1(1.2) | 4(4.7) | 36(42.4) | 44(51.8) | 2.45 | 0.65 |  |
| Select resources to support student learning | 1(1.2) | 4(4.7) | 44(51.8) | 36(42.9) | 2.35 | 0.63 |  |
| Select relevant readings | 1(1.2) | 5(5.9) | 46(54.1) | 33(38.8) | 2.31 | 0.64 |  |
| Develop student assignments | 1(1.2) | 1(1.2) | 38(44.7) | 45(52.9) | 2.49 | 0.59 |  |
| State grading criteria | 1(1.2) | 5(5.9) | 38(44.7) | 41(48.2) | 2.40 | 0.66 |  |
| Develop teaching strategies that promote critical thinking | 1(1.2) | 7(8.2) | 43(50.6) | 34(40.0) | 2.29 | 0.67 |  |
| **Average aggregate score** |  |  |  |  | **2.37** | **0.48** |  |
| **Self-efficacy in instructor behavior and delivery** |  |  |  |  |  |  |  |
| Deliver teaching methodologies | 4(4.7) | 3(3.5) | 42(49.4) | 36(42.4) | 2.29 | 0.75 |  |
| Select and use a variety of teaching strategies | 1(1.2) | 7(8.2) | 44(51.8) | 33(38.8) | 2.28 | 0.67 |  |
| Initiate discussion with students (in class or online) | 2(2.4) | 3(3.5) | 37(43.5) | 43(50.6) | 2.42 | 0.68 |  |
| Draw students into discussions (in class or online) | 2(2.4) | 6(7.1) | 44(51.8) | 33(38.8) | 2.27 | 0.70 |  |
| Communicate at a level that matches student's ability to comprehend | 1(1.2) | 5(5.9) | 44(51.8) | 35(41.2) | 2.33 | 0.64 |  |
| Ask open-ended, stimulating questions | 1(1.2) | 6(7.1) | 42(49.4) | 36(42.4) | 2.33 | 0.66 |  |
| Recognize and respect individual differences | 1(1.2) | 5(5.9) | 32(37.6) | 47(55.3) | 2.47 | 0.67 |  |
| Manage student disagreements with instructor | 0(0.0) | 8(9.4) | 38(44.7) | 39(45.9) | 2.36 | 0.65 |  |
| Communicate consistently both verbally and non-verbally | 1(1.2) | 5(5.9) | 45(52.9) | 34(40.0) | 2.32 | 0.64 |  |
| Show respect for student ideas and abilities | 0(0.0) | 5(5.9) | 23(27.1) | 57(67.1) | 2.61 | 0.60 |  |
| Respond appropriately to students’ questions | 0(0.0) | 2(2.4) | 41(48.2) | 42(49.4) | 2.47 | 0.55 |  |
| Respond to student emotional reactions in class | 0(0.0) | 6(7.1) | 38(44.7) | 41(48.2) | 2.41 | 0.62 |  |
| Integrate readings and teaching methodologies | 9(10.6) | 10(11.8) | 37(43.5) | 29(34.1) | 2.01 | 0.95 |  |
| Initiate discussion with a student with a failing grade | 0(0.0) | 4(4.7) | 46(54.1) | 35(41.2) | 2.36 | 0.57 |  |
| **Average aggregate score** |  |  |  |  | **2.35** | **0.47** |  |
| **Self-efficacy in evaluation and examination** |  |  |  |  |  |  |  |
| Construct exam questions that require integration of content, critical thinking and self-expression | 13(15.3) | 16(18.8) | 35(41.2) | 21(24.7) | 1.75 | 1.00 |  |
| Construct test questions that are at cognitive domain of apply or higher (apply, analyze, evaluate, create) | 16(18.8) | 13(15.3) | 37(43.5) | 19(22.4) | 1.69 | 1.02 |  |
| Develop a test plan | 12(14.1) | 14(16.5) | 34(40.0) | 25(29.4) | 1.85 | 1.01 |  |
| Score exams and interpret results | 11(12.9) | 10(11.8) | 25(29.4) | 39(45.9) | 2.08 | 1.05 |  |
| Evaluate student assignments | 11(12.9) | 8(9.4) | 24(28.2) | 42(49.4) | 2.14 | 1.05 |  |
| Utilize exams as learning tools | 10(11.8) | 15(17.6) | 31(36.5) | 29(34.1) | 1.93 | 1.00 |  |
| Provide constructive feedback on exams and assignments | 11(12.9) | 7(8.2) | 27(31.8) | 40(47.1) | 2.13 | 1.03 |  |
| Identify a student having academic/clinical practice difficulty | 10(11.8) | 11(12.9) | 30(35.3) | 34(40.0) | 2.04 | 1.01 |  |
| Direct or advise students who are experiencing academic/clinical practice difficulty | 11(12.9) | 10(11.8) | 32(37.6) | 32(37.6) | 2.00 | 1.01 |  |
| Conclude a student's clinical practice performance is failing | 11(12.9) | 9(10.6) | 27(31.8) | 38(44.7) | 2.08 | 1.04 |  |
| Utilize self-evaluation to improve teaching | 0(0.0) | 2(2.4) | 37(43.5) | 46(54.1) | 2.52 | 0.55 |  |
| Arrange for constructive feedback and suggestions from peers | 1(1.2) | 4(4.7) | 33(38.8) | 47(55.3) | 2.48 | 0.65 |  |
| Use feedback from students to improve teaching | 0(0.0) | 2(2.4) | 29(34.1) | 54(63.5) | 2.61 | 0.54 |  |
| Evaluate the expected outcomes of a course | 1(1.2) | 4(4.7) | 38(44.7) | 42(49.4) | 2.42 | 0.64 |  |
| **Average aggregate score** |  |  |  |  | **2.12** | **0.72** |  |
| **Self-efficacy in clinical practice** |  |  |  |  |  |  |  |
| Set clinical practice expectations that are appropriate for the level of the learner in patient care areas | 22(25.9) | 15(17.6) | 29(34.1) | 19(22.4) | 1.53 | 1.11 |  |
| Modify clinical teaching strategies based on learner's level of performance | 14(16.5) | 14(16.5) | 38(44.7) | 19(22.4) | 1.73 | 0.99 |  |
| Ask questions in a clinical practice setting that stimulate problem-solving | 14(16.5) | 12(14.1) | 29(34.1) | 30(35.3) | 1.88 | 1.07 |  |
| Provide constructive feedback in a supportive manner regarding clinical practice performance | 17(20.0) | 9(10.6) | 23(27.1) | 36(42.4) | 1.92 | 1.16 |  |
| Demonstrate confidence in the student | 13(15.3) | 9(10.6) | 19(22.4) | 44(51.8) | 2.11 | 1.11 |  |
| Assist student in new patient care situations | 16(18.8) | 10(11.8) | 25(29.4) | 34(40.0) | 1.91 | 1.13 |  |
| Stimulate the student’s interest to learn professional behavior and competence | 17(20.0) | 11(12.9) | 31(36.5) | 26(30.6) | 1.78 | 1.10 |  |
| Adjust clinical practice assignments to individual's level of performance and confidence | 14(16.5) | 9(10.6) | 34(40.0) | 28(32.9) | 1.89 | 1.05 |  |
| Use evaluation criteria to appraise student's clinical practice performance | 15(17.6) | 13(15.3) | 25(29.4) | 32(37.6) | 1.87 | 1.11 |  |
| Record and use anecdotal observations as part of clinical practice evaluation | 16(18.8) | 16(18.8) | 35(41.2) | 18(21.2) | 1.65 | 1.02 |  |
| Integrate best practices into simulation-based experience | 20(23.5) | 13(15.3) | 30(35.3) | 22(25.9) | 1.64 | 1.11 |  |
| Develop expected outcomes for simulation-based experiences | 17(20.0) | 11(12.9) | 34(40.0) | 23(27.1) | 1.74 | 1.07 |  |
| Modify simulation facilitation to student’s level of experience and competence | 20(23.5) | 12(14.1) | 32(37.6) | 21(24.7) | 1.64 | 1.10 |  |
| Use debriefing after a simulation-based experience to encourage learning | 14(16.5) | 12(14.1) | 28(32.9) | 31(36.5) | 1.89 | 1.08 |  |
| Use simulation expected outcomes as basis for student evaluation | 17(20.0) | 14(16.5) | 30(35.3) | 24(28.2) | 1.72 | 1.09 |  |
| Provide a supportive learning environment for the simulation-based experience | 17(20.0) | 12(14.1) | 29(34.1) | 27(31.8) | **1.78** | **1.11** |  |
| **Average aggregate score** |  |  |  |  | **1.79** | **0.95** |  |
| **NC = Not confident; SC = Somewhat confident’, MC = Moderately confident, CC = Completely confident** | | | | | | | |
